# Sleep electroencephalography as a window into future brain health

**DOI:** 10.1093/sleep/zsag152

**Published:** 2026-06-01

**Authors:** Ashley Montero, Andrew Vakulin

**Affiliations:** Flinders Health and Medical Research Institute (FHMRI), Flagship Sleep Health Program, College of Medicine and Public Health, Flinders University, Bedford Park 5042, South Australia; Flinders Health and Medical Research Institute (FHMRI), Flagship Sleep Health Program, College of Medicine and Public Health, Flinders University, Bedford Park 5042, South Australia

Sleep is a fundamental pillar of health and wellbeing. Sleep electroencephalography (EEG) has long been recognized as a marker of brain function [1]. More recently, interest has expanded beyond its traditional role in characterizing sleep architecture and sleep quality, toward its predictive potential as a biomarker of cognitive decline and dementia risk [2]. However, the complexity and high dimensionality of overnight EEG signals with a large number of potential metrics and features being examined in the literature has hindered its translation into more holistic and clinically meaningful EEG markers of cognition. Exciting new research published in this issue of SLEEP by Marino et al. [3] attempted to address this by using a single, EEG-derived predictive metric for late-life cognition.

Dementia prevalence is increasing globally and is major cause of disability and loss of independence; currently, dementia affects an estimated 57 million people globally, but these estimates are expected to triple by 2050 [4, 5]. Identifying earlier predictive biomarkers of later life cognitive decline is a timely priority. Livingston et al. [6] identified that nearly half of all dementia cases could theoretically be prevented by eliminating risk factors in early and midlife. Thus, identifying those who present greatest risk using predictive modeling could guide preventative and risk reduction efforts earlier in life.

Ganglberger et al. [7] have recently developed novel, integrated sleep EEG-derived marker, the Brain Health Score (BHS), using deep learning modeling on cross-sectional cohort data. Building on this work, exciting new findings by Marino et al. [3] identified that the BHS was longitudinally associated with future cognitive function. Specifically, higher BHS values measured in mid-to-late life were associated with better cognitive function more than a decade later across multiple domains including memory, language, executive function, and digital clock drawing. Importantly, the key associations between the EEG Brain Health Score and future cognitive function persisted after adjustment for sociodemographic, behavioral, and health-related covariates as well factors impacting sleep health (sleep duration, sleep efficiency, WASO, sleep apnea, daytime sleepiness, sleep medications, and insomnia symptoms). Taken together, this work underscores the potential of a more holistic sleep EEG marker of brain health and a prospective indicator of future cognitive function.

A strength of this work lies in its departure from traditional discrete EEG feature analysis. Historically, research on sleep EEG and cognition has focused on individual spectral activity, event-related spindles or slow waves, or sleep-stage metrics [8]. While informative, these approaches risk the oversimplification of these complex neural systems. By contrast, the novel utility of BHS condenses the full richness of overnight EEG into a single score via a deep learning approach, making it more appealing for clinical deployment. This shift toward data-driven, composite metrics aligns with broader trends seen in sleep and neurobiological aging research [9–11]. These findings highlight sleep EEG as a powerful marker for predictive brain health and raise the possibility that neural vulnerability can be indexed years before overt cognitive decline becomes detectable.

The implications of this work extend well beyond epidemiology. Future studies and clinical trials are needed to validate these findings in much more diverse populations and settings. Replicable findings would indicate that EEG-derived brain health scores could play a role in early risk stratification for cognitive decline. Sleep studies are already widely conducted in research and clinical settings, and automated EEG analysis offers a non-invasive, low-burden method for identifying individuals at elevated risk years before clinical symptoms emerge. Supplementing sleep EEG with brief in-clinic or follow-up cognitive assessments could provide valuable screening information in general populations and sleep-disordered populations who may be more predisposed to cognitive impairment and decline. Given the widespread ownership of smartphones, the utility of short, at-home cognitive testing administered at regular intervals (e.g. yearly) would be easily implementable. Such data would enable robust characterization of cognitive trajectories at both an inter- and intra-individual level, allowing insight into population and individual trends. Expanding this data would also provide information on normative values and thresholds for cognitive impairment.

Such an approach could be particularly valuable in the context of preventive interventions, where targeting individuals during midlife before older age may yield the greatest benefit. Moreover, a sleep-based biomarker may capture dynamic, modifiable aspects of brain health, raising the possibility that improvements in sleep physiology could translate into improved long-term cognitive outcomes. This hypothesis warrants direct testing.

Despite this novel and valuable contribution to sleep and aging research, several important questions remain. The study sample, while well characterized, is predominantly non-Hispanic White, particularly in the Generation 2 cohort (99%). Ensuring that EEG-derived scores generalize across diverse populations and do not exacerbate existing disparities in dementia risk assessment will be critical as this line of work advances. Meta-analytical data suggest that dementia risk is greater in lower-socioeconomic groups [12]. External validation in more heterogeneous cohorts is therefore an essential next step.

Beyond validation, we need to consider the effect of night-to-night variability in sleep quality and obstructive sleep apnea (OSA), and the implications for the stability of any sleep derived EEG scores and associations with cognitive outcomes. Sleep has high night-to-night variability due to a variety of factors including light, noise, body position, sleep disturbance, or prior sleep loss [13]. Hogan et al. [14] found that the night-to-night standard deviation in the sleep EEG-derived Brain Age Index was 7.5 years. Therefore, it is recommended that multiple nights of monitoring are required for consistency in sleep EEG. This phenomenon is also apparent in sleep disordered populations. There is high night-to-night variability in OSA severity [15], which contributes to sleep fragmentation, ultimately, influencing EEG signals. Lechat et al. [16] suggest that 14 nights of monitoring are needed to accurately assess OSA severity due to night-to-night variability. Meanwhile, data from over 3.7 million person-nights by Leota et al. [17] reveals that estimates of sleep irregularity require 6 to 10 weeks of continuous monitoring. More work is needed to confirm whether the EEG-derived BHS represents a relatively stable profile of brain health or if there is indeed significant intra and inter-individual variability in these EEG biomarkers across multiple nights.

It would be beneficial to explore whether testing of the BHS even earlier in life would show similar strength associations as markers for future brain health in older age. Moreover, the average age of participants studied by Marino et al. [3] was 56 ± 8 years. EEG analysis in younger people who undergo a sleep study may provide insight into cognitive health in later life, but also potential indicators for early onset dementia, a cohort also growing in disease prevalence [18]. If findings are replicable, the critical next step is to determine whether we can implement strategies/treatments to improve sleep, to slow down cognitive decline later in life. This is a long-term research pathway, but necessary to better understand how to optimize cognitive health, promote longevity, and prevent early mortality.

In summary, Marino et al. [3] provide compelling longitudinal evidence that sleep EEG contains prognostic information about future cognitive health. By demonstrating that a single, integrated composite score predicts cognitive performance more than a decade later, this work shifts the field closer to a future in which sleep study recordings contribute meaningfully to early screening to support preventative and risk reduction strategies for managing brain health. Realizing this potential will require replication, methodological refinement, and thoughtful consideration of equity and implementation. Nevertheless, this central message is clear: the sleeping brain carries signatures which may act as a window into future cognitive aging.

## Disclosure statement

*Financial disclosure:* A.M. has no conflicts of interest to declare. A.V. research supported by the National Health and Medical Research Council project grants and fellowship. He has received competitive research funding and equipment from ResMed, Philips Respironics, Withings, and Neuroflex for research unrelated to this manuscript.

*Non-financial disclosure:* A.M. has no conflicts of interest to declare. A.V. is a non-financial Board member of the Australasian Sleep Association and Sleep Health Foundation.
